# Technical and Institutional Factors Affecting Specimen Adequacy and Complications in Ultrasound-guided Kidney Biopsy: A Retrospective Cohort Study

**DOI:** 10.1177/20543581251336551

**Published:** 2025-05-06

**Authors:** Sydney Murray, Chance Dumaine, Chris Wall, Tamalina Banerjee, James Barton, Michael Moser

**Affiliations:** 1 College of Medicine, University of Saskatchewan, Saskatoon, Canada; 2 Department of Medicine, University of Saskatchewan, Saskatoon, Canada; 3 Department of Radiology, Saskatoon Health Region, SK, Canada; 4 Department of Pathology and Laboratory Medicine, Saskatoon Health Region, SK, Canada; 5 Department of Surgery, University of Saskatchewan, Saskatoon, Canada; 6 Saskatchewan Renal Transplant Program, Saskatoon, Canada

**Keywords:** kidney biopsy, ultrasound, complications, technical factors, multivariate analysis, specimen adequacy

## Abstract

**Background:**

Percutaneous ultrasound-guided kidney biopsy is a critical diagnostic tool with a higher rate of complications than most other biopsies. Our prior research identified technical factors that might improve outcomes.

**Objective:**

The objective was to measure the impact of these technical and institutional interventions on specimen adequacy and complication rates in kidney biopsies.

**Design:**

This is a retrospective cohort study comparing outcomes before and after intervention implementation.

**Setting:**

Two hospitals within a single health region in Saskatchewan serving a population of approximately 1 million.

**Patients:**

All adult percutaneous ultrasound-guided kidney biopsies performed on adult patients between 2012 to 2016 (n = 242, pre-implementation) and 2017 to 2021 (n = 338, post-implementation). Both native and transplant biopsies were included, while patients under 18, open biopsies, and biopsies of kidney masses were excluded.

**Measurements:**

Primary outcomes included specimen adequacy and biopsy complications (hematoma, hemoglobin drop, infection, and arteriovenous fistula formation).

**Methods:**

Technical recommendations included introducing the biopsy needle at a 60° angle, targeting a pole, and avoiding the vascular medulla. Institutional recommendations included microscopic screening for all biopsies, limiting the number of radiologists performing procedures, using a checklist, and restricting computed tomography (CT)-guided biopsies to exceptional cases. Multivariate regression analysis assessed biopsy outcomes before and after the recommendations, controlling for known confounders while at the same time refining factors associated with fewer complications and greater diagnostic yield.

**Results:**

The rate of non-diagnostic specimens decreased from 10.3% to 4.4% (*P* = .005), and complications decreased from 35.5% to 14.2% (*P* < .0001). Two or three passes yielded excellent diagnostic success, while 4 passes increased the risk of a complication. Multivariate analysis, after accounting for the collinearity of certain technical factors revealed that medulla avoidance and biopsies done after the implementation of the 2016 recommendations significantly reduced the risk of complications (odds ratio [OR] = 0.37, *P* < .001) and non-diagnostic biopsies (OR = 0.31, *P* = .002).

**Limitations:**

Retrospective design and novelty bias may be a cause of bias in this study. Because the institutional recommendations were followed for all biopsies, it was not possible to distinguish which recommendation was most associated with the improvements. Because our study was done in a single health region, it is not clear if they are generalizable to other programs.

**Conclusions:**

The technical and institutional interventions implemented significantly improved specimen adequacy and reduced complication rates in ultrasound-guided kidney biopsies. We have added to these recommendations in that we have refined the requirement for angling the biopsy needle for ease of use and suggest limiting the number of passes to 2 or 3 whenever possible.

## Abrégé

**Contexte:**

La biopsie rénale percutanée échoguidée est un outil diagnostique essentiel, mais elle présente un taux de complications supérieur à la plupart des autres biopsies. Des recherches antérieures nous ont permis d’identifier des recommandations techniques susceptibles d’améliorer les résultats.

**Objectif:**

Mesurer l’impact de recommandations techniques et institutionnelles sur la qualité des échantillons et les taux de complications des biopsies rénales.

**Conception:**

Étude de cohorte rétrospective comparant les résultats avant et après la mise en œuvre des recommandations.

**Cadre:**

Deux hôpitaux situés dans une même région sanitaire de la Saskatchewan desservant une population d’environ un million de personnes.

**Sujets:**

Ont été incluses toutes les biopsies rénales percutanées échoguidées, natives et post-transplantation, réalisées chez des patients adultes entre 2012 et 2016 (n=242, avant la mise en œuvre) et entre 2017 et 2021 (n=338, après la mise en œuvre). Les biopsies réalisées chez des patients de moins de 18 ans, les biopsies par chirurgie ouverte et les biopsies de masses aux reins ont été exclues.

**Mesures:**

Les principaux critères d’évaluation étaient la qualité de l’échantillon et les complications liées à la biopsie (hématome, chute d’hémoglobine, infection et formation d’une fistule artérioveineuse).

**Méthodologie:**

Introduire l’aiguille de biopsie à un angle de 60°, cibler un pôle et éviter la médulla vasculaire figuraient parmi les recommandations techniques. Exiger un controle microscopique pour toutes les biopsies, limiter le nombre de radiologues effectuant les procédures, utiliser une liste de contrôle et réserver les biopsies guidées par tomodensitométrie aux cas exceptionnels constituaient les recommandations institutionnelles. Les résultats de la biopsie avant et après l’application des recommandations ont été évalués par analyse de régression multivariée en contrôlant les facteurs de confusion connus et en affinant les facteurs associés à une réduction des complications et à un meilleur rendement diagnostique.

**Résultats:**

Le taux d’échantillons non diagnostiques est passé de 10,3 % à 4,4 % (p=0,005) et le taux de complications de 35 % à 14,2 % (p<0,0001). Le fait de procéder en deux ou trois passages a montré un excellent succès diagnostique, alors que le risque de complications a augmenté avec quatre passages. Après la prise en compte de la colinéarité de certaines recommandations techniques, l’analyse multivariée a révélé que l’évitement de la médullaire et la mise en œuvre des recommandations de 2016 réduisent significativement le risque de complications (RC: 0,37; p<0,001) et de biopsies non diagnostiques (RC: 0,31; p=0,002).

**Limites:**

La conception rétrospective de l’étude et un biais de nouveauté pourraient être des sources d’erreur. Les recommandations institutionnelles ont été suivies pour toutes les biopsies, ce qui nous a empêchés de déterminer laquelle était associée aux améliorations. L’étude a été réalisée dans une seule région sanitaire, nos résultats pourraient ne pas être généralisables à d’autres programmes.

**Conclusion:**

La mise en œuvre des recommandations techniques et institutionnelles a grandement amélioré la qualité des échantillons et réduit les taux de complications des biopsies rénales échoguidées. Nous ajoutons à ces recommandations que nous avons affiné l’exigence d’inclinaison de l’aiguille de biopsie pour faciliter son utilisation. Nous suggérons en outre de limiter le nombre de passages à deux ou trois autant que possible.

## Introduction

The percutaneous kidney biopsy remains an important diagnostic test in evaluating someone suspected of having kidney parenchymal disease,^1^ yet they have a higher risk of complications compared to other percutaneous biopsies. Although medical factors for biopsy-related complications are well-defined,^2^ technical factors influencing outcomes have been less thoroughly examined. In our previous study conducted in 2017, we identified key technical factors that can potentially minimize complications and reduce the rate of non-diagnostic specimens.^3^ Based on the results of our study, our multidisciplinary research group composed of nephrologists, radiologists, a kidney pathologist, and a kidney transplant surgeon proposed a set of technical and institutional recommendations (Table 1). The recommendations were supported by our Health Authority and introduced and discussed in multiple forums, including Grand Rounds, Research Days across 3 departments, and at Nephrology and Radiology division meetings. To assess the effectiveness of these recommendations, we compared the biopsy outcomes from our initial study (2012-2016) with those from the current study (2017-2021). In addition, we conducted a multivariate regression analysis on the data from both studies (n = 580) to understand better how technical and institutional factors contribute to complication rates and the occurrence of non-diagnostic specimens. Our objective was to provide data to support the continued use of the recommendations introduced in 2017 and to explore strategies for further optimizing biopsy procedures in the future.

**Table 1.** Recommendations for Percutaneous Renal Biopsies Enacted in 2017.

Category	Recommendations
Technical	1. Introduce the needle into the kidney at an “angle of attack” of ~60°
	2. Target a pole of the kidney
	3. Avoid the vascular kidney medulla
Institutional	1. Mandate on-site immediate screening for glomeruli for all biopsies
	2. Limit the number of radiologists performing renal biopsies
	3. Use of a checklist before each biopsy
	4. Avoid the use of CT scans, except in exceptional cases

## Methods

### Study Design, Setting, and Participants

This is a retrospective study of all ultrasound-guided percutaneous kidney biopsies performed between January 1, 2012, and June 30, 2016, and between June 1, 2017, and May 31, 2021, in 2 hospitals within 1 health region with a catchment of 1 million population. The recommendations for improving the safety and diagnostic accuracy of kidney biopsies were implemented on June 1, 2017, so all biopsies obtained after that date were included in the intervention group. The exclusion criteria for our study included patients under 18 years old, open biopsies, and biopsies of kidney masses. Because computed tomography (CT)-guided biopsies were identified to be associated with a high rate of complications in our previous study and the recommendations included avoiding them, CT-guided biopsies (n = 2) were not included in this study. Both native and transplant kidney biopsies were included in the study.

Dedicated interventional radiologists, all with at least 2 years of prior experience in addition to fellowship training (4 at one site and 3 at the other site) performed all 337 percutaneous ultrasound-guided kidney biopsies. Prior to the implementation of the 2016 recommendations, all interventional radiologists (> 10 at each site) could potentially perform kidney biopsies. Our previous review of kidney biopsies at our center revealed that some interventional radiologists were only performing on average 1 or 2 kidney biopsies per year. We reasoned that concentrating the biopsies to a group of dedicated interventional radiologists (who had an interest in and volunteering to continue doing this challenging and risky biopsy) would help to improve technical skills and minimize complications. This, we felt, was consistent with the literature in both the radiological and surgical literature that support improved outcomes seen with higher operator volumes.^4^

After June 1, 2017, a team verification checklist was used for all patients, ensuring a pathologist or technician was present for microscopic screening, the angle of attack (AOA) was 60° with a polar approach, and blood pressure was below 160/90. Anticoagulation, including partial thromboplastin time (PTT), international normalized ratio (INR), and platelet count, was also checked. We followed the 2019 SIR guidelines for high-risk percutaneous procedures, holding or bridging anticoagulation as required based on the specific drug.^5^

Using ultrasound, the kidney was visualized in a longitudinal plane close to the middle of the anterior-posterior diameters. The needle was advanced and visualized in real-time, then backed up and adjusted as needed. When the needle was in a satisfactory position, an automatic spring-loaded device (15-, 16- or 18-gauge) with a core length of 2.2 cm was used to obtain the biopsies. Two or more cores were obtained for each patient. All biopsy samples were sent to a single center to be processed and read by one of 3 fellowship-trained kidney pathologists.

### Data Collection

**Figure 1.**
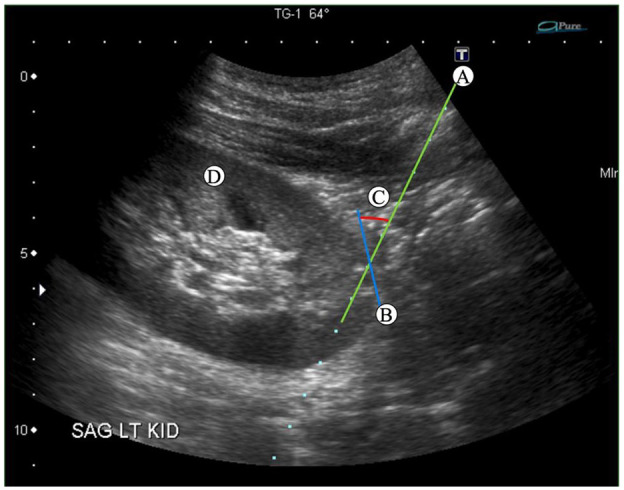
Ultrasound image taken at the time of biopsy. (A) Biopsy needle, (B) tangential line, (C) angle of attack (AOA), and direction of needle in relation to the medulla (D) kidney.

Clinical data on biopsy complications was obtained from patient medical records. Pathology reports were consulted to document specimen adequacy and diagnosis. Technical data for each biopsy were collected from digitally achieved images and reports within our center’s Picture Archiving and Communication System (PACS). The image taken at the time of the first biopsy was printed on paper. A line was drawn tangential to the point of entry into the kidney, and the angle between this line and the needle was recorded as the AOA (Figure 1). The depth of the biopsy needle (from the skin to the point where the needle enters the kidney) was measured based on the legend on the lateral edge of the ultrasound image. To determine if the medulla was targeted or avoided, we assessed the direction of the biopsy needle in relation to the medulla. If the biopsy needle or a continuation of the line of the biopsy needle entered the medulla, it was considered to have targeted the medulla. To determine the orientation of the biopsy needle in relation to the hilum, the continuation of the biopsy line was used. Biopsy needles directed away from a line drawn perpendicular to the long axis of the kidney at the midpoint of the kidney were classified as “away from hilum.” Biopsy needles aimed toward this line were classified as “towards the hilum.”

This research was approved by the University of Saskatchewan Research Ethics Board (BioREB#4049) and the Saskatchewan Health Authority.

### Outcomes

Outcomes of interest included the adequacy of the biopsy specimen and biopsy complications. Adequacy outcomes included the number of glomeruli per core and whether the specimen was adequate for making a pathological diagnosis. Cases where only 3 or fewer glomeruli were seen were considered inadequate due to the need to subdivide the core into 3 different sections (light microscopy, immunofluorescence, and electron microscopy). In cases where the diagnosis was unclear to 1 kidney pathologist, the case was reviewed with a second kidney pathologist. A biopsy was classified as non-diagnostic if a diagnosis could not be ascertained by 2 specialized kidney pathologists. If a diagnosis could be made, then a biopsy was considered adequate.

Complications recorded included a bleed reported on ultrasound, a large hematoma greater than 5 cm, a hemoglobin drop greater than 10 g/L, infection (cellulitis or abscess), and arteriovenous (AV) fistula formation.

### Statistical Analysis

Chi-square testing was used to compare incidences in 2 or more groups, while the Mann-Whitney U test was used to compare the number of glomeruli per core.

Multivariate models of factors associated with complications, specimen adequacy, and glomeruli per core were constructed as suggested by Reboldi et al^6^ based on technical and institutional factors previously shown to be related to the outcomes. Because there were few large hematoma complications and few pseudoaneurysms resulting from biopsies, “complications” represented a composite of all the recorded complications as in our prior publication on the subject.

Technical factors of AOA, whether the biopsy needle was toward or away from the hilum and whether the medulla was targeted or avoided, were highly correlated and could not be used in the same model due to collinearity. Complications correlated most strongly with medulla targeting and AOA in univariate analysis. Therefore, separate models were constructed for medulla-avoiding and for biopsies done with an AOA of 50 to 70°. The number of passes, as identified in univariate analysis, was included in the multivariate model. Whether needle gauge is a factor in complications is not yet clear based on prior studies. Because we observed *P*-values of less than 0.2 in our prior study, we included this variable as well. Finally, in the study design, we decided that biopsies of transplant kidneys should be controlled for, given that they are often performed to detect rejection in kidneys with otherwise normal parenchyma. In addition, the scar tissue capsule that forms around a transplanted kidney differs significantly from the Gerota’s fascia surrounding a native kidney.

Institutional factors included having a microscopic screening for all biopsies, limiting the number of radiologists performing kidney biopsies, using a checklist before each procedure, and avoiding CT scans for kidney biopsies except in special cases (n = 2, excluded from this study). Starting June 1, 2017, on-site immediate screening for glomeruli was performed for every biopsy. The variable “After 2016 recommendations” was therefore used to encompass all 4 factors.

SPSS Ver 26 (IBM Corporation, Armonk, New York) was used to perform all statistical analyses, including the Mann-Whitney U test, the chi-squared test, and logistic regression analysis. A *P*-value less than 0.05 was considered significant in all cases.

## Results

### Demographics

Table 2 shows the demographics of the biopsies before and after the implementation of the 2016 recommendations. The apparent increase in transplant biopsies reflects a shift to more procedures being performed in the radiology department, as fewer were done with bedside ultrasound outside of radiology. Although ultrasound is used for bedside biopsies, those performed outside the radiology department by a single senior transplant nephrologist were excluded from the study due to the absence of a mechanism to save the screen image during the procedure. The difference in the side biopsied is explained by 2 radiologists who do a large number of kidney biopsies setting up their procedures for native kidney biopsy on the left side. Significant improvements were seen in several measures of specimen adequacy and reduced complications.

**Table 2.** Characteristics of the Ultrasound-guided Biopsies Conducted Before and After the Recommendations Were Enacted.

		Before recommendations (n = 242)	After recommendations (n = 338)	*P*
Demographics	Patient age median (IQR)	49 (34, 62)	55 (38, 66)	.09
	Sex (M:F)	135:107	192:145	.77
	Native: transplant biopsy	197:45	239:99	.**003**
	Side (R:L)	116:126	88:249	**<.001**
Technical factors	Pole (upper: middle:lower)	10:43:189	26:12:294	**<.001**
	Needle size <=16G:>=18G	82:160	14:324	**<.0001**
	Median Angle of Attack (IQR)	73°(58, 83)	65° (50, 78)	**<.0001**
	Medulla-avoiding	122 (50%)	217 (64%)	.**0007**
	Median depth from skin (cm, IQR)	N/A	3 (2, 4)	N/A
	Number of passes	3 (2, 3)	3 (2, 3)	1
Adequacy of specimen	Median number of cores per bx	3 (2, 3)	3 (2, 3)	1
	Median glomeruli per core	8 (5, 11)	11.3 (8.9, 15.5)	**<.0001**
	Non-diagnostic specimen	25/242 (10.3%)	15/338 (4.4%)	.**005**
	Repeat biopsy required	8/242 (3.3%)	3/338 (0.8%)	.**036**
Complications	Bleed on ultrasound image	70 (28.9%)	47 (14.2%)	**<.0001**
	Bleed > 5 cm diameter	15 (6.2%)	9 (2.7%)	.**035**
	Hgb drop >10	28 (11.6%)	10 (2.9%)	**<.0001**
	Bleed on ultrasound image and Hgb drop >10	10 (4.1%)	8 (2.4%)	.24
	AV fistula in kidney	2 (0.1%)	3 (0.1%)	.48
	Total Complications	86 (35.5%)	48 (14.2%)	**<.0001**

Bolded *P* values indicate statistical significance (*p* < 0.05).

### Univariate Analysis of Technical Factors

**Figure 2.**
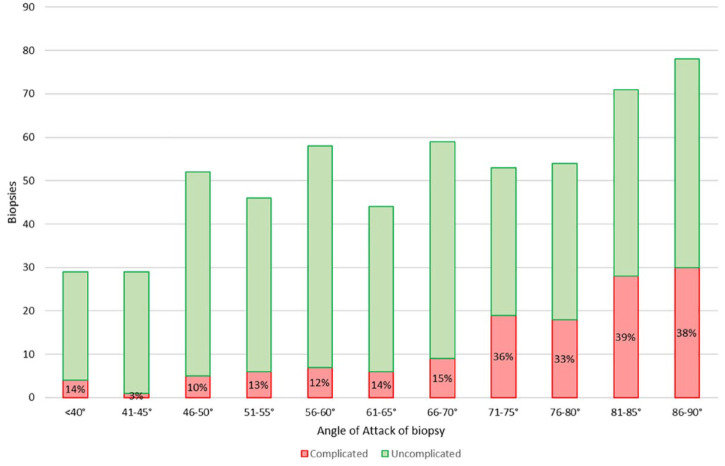
Angle of attack for complicated (red) and uncomplicated (green) percutaneous kidney biopsies (n = 580).

Figure 2 shows the rate of complications for various angles of attack, in keeping with the importance of the AOA documented in our previous publication (Table 3). The AOA, avoiding the medulla, and aiming away from the hilum are highly correlated; because of this, they cannot be entered simultaneously into a multivariate regression model. Complications significantly increased with 4 or more passes of the biopsy needle, so we included this factor in the multivariate analysis. The depth of the biopsy did not correlate with the distribution of the AOA or complications (data not shown). In our previous study, needle size was not a significant factor; however, in the current study (Table 4), the use of a smaller 18-gauge needle for biopsy was associated with significantly fewer complications (20% vs 37%, *P* = .0002). In contrast, the larger 16-gauge or larger needles did not result in superior outcomes regarding the number of glomeruli per core (*P* = .47) or the diagnostic yield of biopsies (*P* = .57).

**Table 3.** Summary of Factors Analyzed Before Developing the Multivariate Analysis Model.

3(a) Correlation of Angle of Attack, Medulla-avoiding, and Needle Angled Away From the Hilum.			
	Medulla-targeting	Medulla-avoiding	*P*
AOA < 50°	4 (4%)	104 (64%)	
AOA 50-70°	67 (31%)	149 (69%)	<.0001
AOA >70°	184 (72%)	72 (28%)	
Needle toward hilum	223 (63%)	130 (37%)	<0.0001
Needle away from hilum	32 (14%)	195 (86%)	
3(b) Number of Passes Done in 1 Biopsy Procedure: Complications and Non-diagnostic Biopsies.			
	N	Complications	Non-diagnostic biopsy
2 passes	224	49 (22%)	18 (8%)
3 passes	253	48 (19%)	17 (6.7%)
4 or more passes	103	35 (34%)	5 (4.9%)
*P*		.01	.892

AOA refers to the angle of attack.

**Table 4.** Multivariate Logistic Regression Analysis of Variables Associated With Complications and Non-diagnostic Biopsies in Percutaneous Ultrasound-guided Renal Biopsies. (Bolded *P* values indicate statistical significance (*p*<0.05))

4(a) Complications.			
Variable	*P*	Odds ratio	95% CI
Medulla-avoiding	**<.001**	0.37	0.24, 0.57
Transplant kidney	.**002**	0.38	0.21, 0.7
After 2016 recommendations	.**002**	0.49	0.31, 0.78
Three or fewer passes	.065	0.62	0.38, 1.03
18G needle or finer	.1	0.64	0.38, 1.1
4(b) Non-diagnostic Biopsies.			
Variable	*P*	Odds ratio	95% CI
Medulla-avoiding	.**002**	0.31	0.15, 0.64
After 2016 recommendations	.**03**	0.45	0.22, 0.93
Three or fewer passes	.35	1.56	0.62, 3.94
18G needle or finer	.66	1.21	0.51, 2.9
Transplant kidney	.91	1.05	0.47, 2.3

### Multivariate Analysis of Complications

The multivariate analysis of complications showed that avoiding the medulla and biopsies performed after the 2016 recommendations were enacted were associated with a lower risk of complications (Table 4). There was also a trend suggesting that using a smaller needle (*P* = .063) and limiting the procedure to 3 or fewer passes (*P* = .075) could be protective. Similar results were seen when using an “angle of attack of 50-70 degrees” instead of medulla avoidance (data not shown).

### Multivariate Analysis of Non-diagnostic Biopsies

As with the analysis of complications, 2 separate models were developed for the collinear variables “medulla-avoiding” and “angle of attack of 50-70 degrees” (Table 4). Biopsies that avoided the medulla (*P* = .002) and those performed after the 2016 recommendations were implemented (*P* = .03) were associated with a reduced risk of non-diagnostic biopsy. The number of passes, needle size, and whether the biopsy involved a transplant or native kidney were not associated with the rate of non-diagnostic biopsy. A multivariate analysis that included an AOA of 50-70 degrees produced very similar results (data not shown).

## Discussion

### Principal Findings

Our study demonstrates that implementing institutional recommendations and technical considerations identified in our previous work reduced non-diagnostic specimens and complications of percutaneous kidney biopsy. The rate of non-diagnostic specimens decreased from 10.3% to 4.4%, and total complications declined from 35.5% to 14.2%, each representing a reduction of more than 50% following the implementation of the recommendations.

Institutional factors included on-site microscopic screening for all biopsies, limiting the number of radiologists doing kidney biopsies, using a checklist before each kidney biopsy, and avoiding the use of CT scanning for kidney biopsies except in extreme cases.

In the current study, all patients had a minimum of 2 passes of the biopsy needle, but after 4 passes, the rate of complications increased significantly. Both 2 and 3 passes provided excellent diagnostic yield, whereas 4 passes were associated with a higher complication rate. Because the order of the cores was not recorded, we cannot provide a detailed breakdown of diagnostic yield per pass. However, it can be said that a fourth pass was possibly obtained for 2 reasons: either the microscopic screening for glomeruli deemed the initial samples insufficient, or some radiologists routinely took 4 cores to avoid a non-diagnostic biopsy. We therefore do not recommend routinely performing 4 passes unless the microscopic screening determines additional sampling is necessary.

Needle size has been a topic of ongoing debate.^7,8^ Although our previous study did not demonstrate significant differences, univariate analysis in the current study revealed that the 18-gauge needle was associated with fewer complications without compromising the diagnostic rate. However, this difference was not statistically significant on multivariate analysis. Given the comparable diagnostic yield of the 16-gauge needle and its potential for increased complications, our radiologists favor the 18-gauge needle for kidney biopsies. We incorporated these factors into multivariate analyses of specimen adequacy and complications. Because of collinearity, an AOA of 50 to 70° and avoiding the medulla could not be included in the same model. In the current study, when controlling for other factors in the model, avoiding the medulla and an AOA of 50 to 70° were associated with a lower rate of non-diagnostic specimens and complications.

### Study Strength and Weaknesses

Our study’s limitations include the potential influence of novelty bias and the Hawthorne effect.^9^ These biases could have contributed to the observed improvements in outcomes, as the introduction of a new approach and awareness of case monitoring might have enhanced performance. However, when we analyzed biopsies performed after 2017, including during the COVID-19 era, we found no significant differences in adherence, complications, or rates of non-diagnostic specimens from year to year. This consistency suggests that, at least early on, the observed improvements are durable and not merely a result of initial novelty or increased scrutiny. In the longer term, the key institutional changes—checklist use, mandatory on-site microscopic screening, and limiting CT-guided biopsies—have become part of the expected routine for all kidney biopsies for nearly a decade, with no signs to suggest reverting to prior practices. The results of this current study have been presented at Grand Rounds and Research Days, and this has been a refresher for the recommendations and a chance to educate on the subject of more flexible approach to the 60° “angle of attack.” While we are happy with the sustained improvements, ongoing surveillance, periodic review, and continued education will be important to maintain adherence.

In the multivariate analysis, technical factors explained some of the variance, yet the post-2016 biopsy period also independently predicted improved outcomes. We believe this variable captures the cumulative effect of the institutional changes. But because every biopsy post-2016 was performed with with on-site microscopic screening, a standardized checklist, a dedicated group of interventional radiologists, and without CT guidance, it is not possible to say which component of the institutional factors played the most important role in the improvement. Our work was, however, further confirmation and refinement of the importance of technical factors as found in our previous study while acknowledging that institutional factors are also important.

Finally, our findings come from a single health region. While key steps, such as avoiding the medulla and using a checklist, can be applied anywhere, not all sites have the staff to assign interventional radiologists to kidney biopsies or have a pathologist or technician at every procedure. Because of the unique challenges of the medical kidney biopsy, smaller centers with fewer cases may consider sending these cases to larger referral centers to ensure the right staffing and experience.

### Comparison With Prior Studies

Our study is in keeping with prior work establishing the value of checklists in procedures and the operating room.^10^ Our inquiry into biopsy protocols at several large Canadian programs found little in the way of formal guidelines to compare to. PubMed, Internet, and AI inquiries also yielded very little on the subject. One Canadian study asked “What is the gold standard?” for kidney biopsy;^11^ while it covered complications, medical risks, and prevention, its procedural recommendations were limited to needle selection. There was no mention of checklist use, microscopic screening microscopic screening, ultrasound versus CT guidance, or interventional radiologist experience.

BC Renal has published kidney biopsy guidelines that include patient assessment, preparation, and a general overview of technique.^12^ However, they do not specify details on pole targeting, checklist use, or most of the technical and institutional factors we implemented.

Other guidelines may exist, but they might be behind institutional firewalls or rarely accessed by physicians who may not even be aware of them. Some centers may already follow similar practices—such as using a checklist or limiting procedures to a small group of experienced interventional radiologists—but these details are not outlined in widely available protocols.

When interviewed, the interventional radiologists performing kidney biopsies within our health region indicated there was difficulty and confusion in determining the AOA in relationship to the kidney. The basic software that came with the ultrasound machine did not lend itself well to determining where the tangential line would be drawn in order to calculate the AOA during the biopsy. Although many radiology reports indicated that the biopsy needle was angled at approximately 60°, our measurements showed that an angle between 50° and 70° was achieved only about two thirds of the time. Radiologists agreed that avoiding the medulla by angling the needle away from perpendicular to the kidney capsule was more achievable. This approach minimizes medullary penetration, if any, by reducing penetration depth, which may be the key to enhanced safety. In rare cases where a medullary sample is required, this approach still ensures a safer biopsy.^3^

### Implications for Clinicians

The findings of this study suggest that incorporating specific technical modifications (angling the biopsy slightly with respect to the kidney, targeting a kidney pole, avoiding the vascular medulla, and limiting biopsy passes to a maximum of 3) and institutional modifications (restricting biopsies to a standardized team of radiologists and ensuring on-site microscopic screening) into ultrasound-guided kidney biopsy protocols can improve diagnostic yield while reducing complications.

### Future Research Directions

Future work should assess the understanding of the recommendations by the radiologists at each of the 2 sites. Although major improvements were made after 2017, there were still a significant number of biopsies where the medulla was targeted (most of these presumably unnecessarily) and could have been avoided. Educational Rounds and other educational activities are being planned around this subject. Clear communication between the nephrologist ordering the biopsy and the radiologist performing the biopsy is essential.

## Conclusions

Results support the institutional recommendations of microscopic screening at every biopsy, using a checklist, avoiding CT-guided kidney biopsies, and limiting the number of interventional radiologists involved. On the technical side, we recommend using an 18-gauge biopsy needle, avoiding the medulla unless necessary to confirm the diagnosis, and angling the needle slightly to avoid a perpendicular entry into the kidney (the ideal is around 60°). We also suggest limiting the number of passes to 2 or 3 whenever possible. Continuing to focus on these strategies and implementing these recommendations at other institutions should improve patient safety and reduce non-diagnostic biopsies in the future.
